# Draft Genome Sequence of *bla*_NDM-1_-Positive *Escherichia coli* O25b-ST131 Clone Isolated from an Environmental Sample

**DOI:** 10.1128/genomeA.00462-14

**Published:** 2014-05-29

**Authors:** Kirthi K. Kutumbaka, Sukkyun Han, James Mategko, Cesar Nadala, Genevieve L. Buser, Maureen P. Cassidy, Zintars G. Beldavs, Scott J. Weissman, Karim E. Morey, Robert Vega, Mansour Samadpour

**Affiliations:** a Institute for Environmental Health (IEH), Lake Forest Park, Washington, USA; b Oregon Public Health Division, Portland, Oregon, USA; c Division of Infectious Diseases, Seattle Children’s Research Institute, Seattle, Washington, USA; d Oregon State Public Health Laboratory, Hillsboro, Oregon, USA

## Abstract

A multidrug-resistant NDM-1 carbapenamase-producing *Escherichia coli* sequence type 131 (ST131) organism was obtained from vacuum cleaner dust collected from the home of a case patient. Here, we report the assembly and annotation of its genome.

## GENOME ANNOUNCEMENT

The emergence of *Escherichia coli* strains resistant to carbapenems is a major public health problem, since these antibiotics are one of the last-line agents for many infections caused by these bacteria (1). Organisms of *E. coli* sequence type 131 (ST131) are global pathogens belonging to the extraintestinal pathogenic *E. coli* lineage. The ST131 lineage is known for CTX-M extended-spectrum β-lactamases (ESBLs) and fluoroquinolone resistance, and it is often associated with urinary tract infections and septicemia (2–5). An ST131 isolate producing New Delhi metallo-β-lactamase-1 (NDM-1) was previously reported (6, 7).

The analytical portion (50 g of vacuum cleaner dust collected from the home of a case patient) was preenriched at 42°C for 2 h in M1 medium (Pi Biologique, WA) without selective agents. Selective incubation was continued by adding 4 mg liter^-1^ of meropenem as a final concentration. The enriched culture was streaked after 24 h on MacConkey agar with antibiotics in order to isolate carbapenem-resistant *E. coli*. The isolate was then confirmed to be NDM-1 positive by an NDM-PCR screening system (Pi Biologique), and the genomic DNA was prepared using the Bacterial DNA purification kit (Pi Biologique) for MiSeq genome sequencing. NDM-1-producing *E. coli* O25b-ST131 was deposited in the IEH culture collection as IEH71520.

The genome library was prepared using the Nextera XT DNA sample prep kit (Illumina, CA), and genome sequencing was performed using the Illumina MiSeq desktop sequencer (Illumina) loaded with a paired-end 2 × 250 cycle MiSeq reagent kit version 2. The raw shotgun reads were then assembled using the A5 assembly pipeline (8), and annotation was done using the RAST server (9). The genome of the isolate has a total length of 5,153,432 bp, with a G+C content of 50.79%, consisting of 202 contigs with an *N*_50_ of 67,135 bp and a maximum contig size of 191,471 bp. A total of 5,123 open reading frames (ORFs) were identified, 98 of which are RNAs, and the rest are protein-coding sequences. BLAST analysis of the contigs identified three complete plasmids and one incomplete plasmid. The plasmids pJJ1886_1, pJJ1886_2 from strain *E. coli* JJ1886 (10), and the *bla*_NDM-1_-encoding plasmid pTR4 (11) had 100% BLAST query coverage. The plasmid pKF3-140 from *Klebsiella pneumoniae* (12) had >80% BLAST query coverage.

The nucleotide sequences of the housekeeping genes *adk*, *fumc*, *gyrB*, *icd*, *mdh*, *purA*, and *recA* were submitted to the multilocus sequence typing database (MLST) (http://mlst.warwick.ac.uk/mlst/dbs/Ecoli) (13) to determine the sequence type (ST), which was identified as ST131. The O25 type of the IEH71520 strain was identified as O25b using the PCR-specific *pabB* allele (14). The variant of the CTX-M enzyme in this strain was found to be CTX-M-27, whereas the majority of the ST131 isolates contain the CTX-M-15 enzyme (5).

IEH71520 is the first draft genome sequence of an NDM-1-positive CTX-M-27-producing *E. coli* O25b-ST131 isolate. In contrast to previously isolated NDM-1-positive *E. coli* isolates, IEH71520 was isolated from the home of a case patient, and therefore, the emergence of this strain is a cause of serious concern, as it has a unique combination of resistance and virulence.

### Nucleotide sequence accession number.

The *E. coli* IEH71520 genome sequence was deposited in GenBank under the accession no. JJNV00000000.
